# Drainage for Health

**Published:** 1886-11

**Authors:** 


DRAINAGE FOR HEALTH; OR, EASY LESSONS IN SANITARY SCIENCE. *By* JOSEPH WILSON, M.D. *Second Edition. Pp. 74. Philadelphia:* P. BLAKISTON, SON & CO., *1886.*

In chapter I, on "Land Drainage," and II, on "Drainage of the Farm House and of the Village," the author discusses the questions of drainage, for the farm and farm house, in so simple and complete a manner, that no reader can fail to understand both the principles involved and the details necessary to carry them out. The idea that the drainage of pine forests is due to the peculiar manner in which pine stumps decay is novel, but put in a very convincing manner.

The author claims that a 2-inch circular drain-pipe is the best form to use, and that it is large enough for the main in a piece of land containing 64 acres. In land-drainage the elliptic and oval forms of pipe "have no advantages which compensate for the additional labor of placing them."

The suggestion, that the upper five feet of the wall of a well should be finished in hydraulic cement, and narrowed "so as to leave an opening only large enough for the pump and a man-hole," is excellent.

The chapter on the drainage of cities is suggestive and practical. The author claims that in following the example of Paris and London, we follow in such a way that we fail to take advantage of the most recent experience of those cities.

The form commended as the best for large sewers, is that in use in Paris. "This has a floor of masonry with a deep channel for ordinary drainage, and there is the arch over it of sufficient capacity for the storm-water." "The best form for smaller sewers is the oval, broad above and narrow below. The floor and lower part to be as water-tight as stones and hydraulic cement can make them, the bricks of the upper part to be also laid in cement." "For the smallest sewers glazed earthenware pipes of the circular form are the best, on account of the smoothness of interior surfaces, and the facility of placing them."

The author condemns the old style of water-closet with the tilting pan-valve, and recommends the simple porcelain hopper closet with hard-rubber floating valves.

The remarks about sinks, and the ventilation of sewers are in accord with the most advanced opinion of sanitary engineers.

The digression in the way of an account of the natural history of the common tape-worm, may serve to deepen the impression of the importance of the subject of stercoral pollution of rivers, at least in the minds of medical men.

The author thinks that "a manufactory of paris green, or the like, might dangerously poison a stream of water; but the organic germs of disease, amœba and bacteria, trichina and filaria, bilharzia and strongylus, cysticercus, echinococcus, tape-worm, and the like, could hardly pass through the dyer's vats and live. The danger from manufactories is from the sewage of their villages."

The way in which the subject of plumbing is treated is odd. The conclusions reached are, 1: "All kinds of leaky pipes of moderate size, may be instantly repaired by giving them a coat of paint and a wrapping of twine; if a small piece of pipe is missing, a piece of gum hose will temporarily answer to replace it and complete the circuit." 2: "A very small leak in a water-closet, such as no plumber discovers in a common inspection, may waste as much water as a hundred families can actually use." 3: "About two-thirds of all the water pretended to be used in our cities, is mere waste through defective plumbing, so concealed as not to be found in the ordinary inspections." This last statement is based upon the experience of a naval hospital.

The concluding chapter, "Plea for Board of Health," is an account of the experience of a house owner, living in a block of buildings, who found in dealing with the Board of Health that the members were incompetent men in their position. The author thinks that is often true, and that such boards should be composed only of men who practically understand sanitary science.

The author's literary style is at times very odd and prolix, but the book as a whole is excellent and is accompanied by a good index.

